# De-Bonding Numerical Characterization and Detection in Aeronautic Multi-Element Spars

**DOI:** 10.3390/s22114152

**Published:** 2022-05-30

**Authors:** Antonio Concilio, Monica Ciminello, Bernardino Galasso, Lorenzo Pellone, Umberto Mercurio, Gianvito Apuleo, Aniello Cozzolino, Iddo Kressel, Shay Shoham, David Bardenstein

**Affiliations:** 1 Adaptive Structures Division, the Italian Aerospace Research Centre (CIRA), 81043 Capua, Italy; m.ciminello@cira.it (M.C.); b.galasso@cira.it (B.G.); l.pellone@cira.it (L.P.); u.mercurio@cira.it (U.M.); 2 Research Division, Piaggio Aerospace Industries, 81043 Capua, Italy; gapuleo@piaggioaerospace.it (G.A.); acozzolino@piaggioaerospace.it (A.C.); 3 Advanced Structural Technologies, Engineering Center, Israel Aerospace Industries (IAI), Ben Gurion International Airport, Tel Aviv 70100, Israel; ikressel@iai.co.il (I.K.); sshoham@iai.co.il (S.S.); dbardenstein@iai.co.il (D.B.)

**Keywords:** adhesive bonding, composite structures, sensors, damage characterization, smart devices, structural health monitoring

## Abstract

Structural health monitoring has multifold aims. Concerning composite structures, the main objectives are perhaps reducing costs by shifting from scheduled to on-demand maintenance and reducing weight by removing redundant precautions as the insertion of chicken fasteners to for ensuring joint safety in cases of bonding layer fail. Adhesion defects may be classified along different types, for instance distinguishing between glue deficiency or de-bonding. This paper deals with a preliminary numerical characterization of adhesive layer imperfections on a representative aircraft component. The multipart composite spar is made of two plates and two corresponding C-beams, bonded together to form an almost squared boxed section beam. A numerical test campaign was devoted to extract relevant information from different defect layouts and to try to assess some parameters that could describe their peculiarities. A focus was then given to macroscopic evidence of fault effects behavior, as localization, reciprocal interference, impact on structural response, and so on. A proprietary code was finally used to retrieve the presence and size of the imperfections, correlating numerical outcomes with estimations. Activities were performed along OPTICOMS, a European project funded within the Clean Sky 2 Joint Technology Initiative (JTI).

## 1. Introduction

Composites are acknowledged as appealing materials that can be tailored to specific necessities; in spite of that, there are many issues that have hampered their full development in aerospace. For instance, their acoustic and vibrational behavior needs improvement, which requires the use of large masses of absorbers [1,2]. Generally, apart from intrinsic weaknesses that can be overcome by a suited design, there are aspects directly correlated to their technological development.

In particular, the damage of composites deserves attention [3,4]. For instance, the defect is usually hidden within their body and may become evident only as the structural system is close to collapse [5]. Low-energy impacts such as the ones correlated to hail, FOD (foreign object damage), and bonding-associated flaws may be hardly detectable and may lead to significant uncertain variations in the structural behavior. For instance, low-energy hits usually produce barely visible damage (BVID) or invisible on the surface, while they generate a deep delamination. This event can be, in turn, associated with local fiber breakdown, and even the detachment of components. Such considerations have historically led the authorities to require conservative safety measures, which have resulted in the structure oversize, for instance, by increasing the components’ thickness and the number of mechanical connections [6].

These two outcome examples are driven by the necessity of ensuring the structure with a certain residual capability of bearing operational loads or inhibiting crack propagation. In synthesis, a fail-safe approach is implemented by retarding the occurrence of catastrophic events, which ensures the probability of individuating the damage long before the collapse occurs. However, this approach deeply reduces (and may even cancel) many of the outstanding advantages the composite exhibits with respect to traditional structures. Additionally, these precautions further complicate the maintenance process, which should be performed by highly specialized personnel [7,8].

It is therefore necessary to explore solutions that could allow overcoming current showstoppers. Structural health monitoring (SHM) technology has been developed per years with the established target to provide the architectural skeleton with a diagnostic capability to understand its integrity level (or, health state) [9]. Prognostics functions are currently under study, even if their development stage is far less mature [10,11].

Simple SHM systems have matured enough to be operated in operational aeronautic environment. For instance, confined sensor networks with dedicated elaboration data algorithms are used to detect flaws in certain high-risk regions (so-called hot-spots) [12,13], such as aircraft door panels and door-frames. In fact, they are continuously hit by automatic or semi-automatic ladders at the ground; they even suffer impacts during closure and opening operations [14,15].

Component bonding safeguarding is a critical factor for assessing composite structural systems’ performance to a suitable level of confidence. Then, monitoring the adhesive layer state may be a key point in assessing its use as a consolidated process. A target specification is, however, needed, since there are at least two different phenomena that could be considered. The first one is concerned with de-bonding, as adhesive layers weaken with operations (fatigue) and the action of external factors (aging). Operations can be further articulated in classical working phases and non-favorable events such as impacts produced by hail, or anything connected to ground processes [16]: the former can be predictable, since it is connected to scheduled missions, while the latter is unpredictable, as it is associated with accidents, even if limited. Both of them are usually treated by statistic models. The second one is instead related to manufacturing imperfections leading to surface irregularity, which cannot be taken completely under control because of several issues [17]. Two structural elements facing each other could, therefore, not have any bonding layer between them for the unevenness of the respective shapes. *Kissing bond* is usually the locution associated with such a circumstance, featuring elements that touch each other directly. It is difficult to detect using NDI (non-destructive inspections) such as the classical C-Scan or even other non-invasive techniques [18,19].

Bonding monitoring shall hence consider the segment of interest for revealing damage insurgence after operations and the bad quality of the adhesion correlated to production defects. Despite the similarity, these sides of the same coin are very different and lead to separate technology developments. In this paper, kissing bonds are specifically addressed.

In the literature, the absence of adhesive layer segments is investigated by various kind of sensor networks. Such a kind of flaw has the undesirable characteristic of giving rise to a very limited effect in the region of persistence. So, single-point arrays show significant limitations; layout and sensor density are critical and should cover any possible affected point. Since the extinction distance is in the range of centimeters, it follows that a 100 cm^2^ area would require about 10 to 20 sensors. For this reason, such an approach is unsuitable unless specific zones are addressed, subjected to a high probability of being corrupted (the already-cited hotspots). In this case, a basic knowledge of the structure and the operation conditions has to be available a priori, allowing the identification of the most sensible regions by design outcomes, experience, experimental evidence, and so on.

Distributed sensor arrays are capable of monitoring significant lengths quasi-continuously and provide a formidable tool to provide detailed info on the reference structure state, even in real-time: their output is comparable to numerical analyses. Data shall be then elaborated through a devoted algorithm for individuating the damage locations; targeted FE models shall be designed with the twofold aim of simulating the phenomena under investigation and replicating sensor acquisitions. Both these tools permit us to create a dedicated methodology for the assessment of a specific SHM system.

## 2. Aims and Motivations of the Study

This paper focuses on the FE representation of the effect of the adhesive layer continuity interruption in terms of strain field modulation. Deformation data are retrieved along a single line, simulating the presence of a quasi-continuous fiber optic. The authors considered that a systematic analysis of the effects of bonding flaws on the structural response is rare in the literature, and therefore, it is believed that the preliminary work herein presented could be of some interest for researchers dealing with this topic and could serve as a basic reference for further and deeper investigations, as well as for the further enhancement of dedicated SHM systems design.

The reference test article is a characteristic wing box element of a small aircraft, made of two symmetrical C-beams, connected through a top and a bottom plate. The four carbon-composite elements are bonded to each other to give rise to an almost square box frame. During manufacturing, bonding interruptions were imposed at the interfaces between C-beam caps and the panels at specific locations. Distributed optical fibers were then embedded between the beam caps and the plates and underwent the curing process together with the rest of the structure. These sensors were able to catch strains with mm density and a threshold lower than 10 microstrains. Static and quasi-static acquisitions were performed.

Our numerical analysis aims at replicating the information attained by the distributed sensor system. Discontinuity in the bonding layer is simulated by removing the bonding elements in the affected regions completely, equivalent to the option of associating them with an almost zero stiffness [20]. A large numerical campaign is then conducted, articulated in two phases. The first one is carried out on a simple component and analyzes the sensibility of the sensor line to irregularities occurring around its proximities in order to obtain a first estimation of the extinction distance. The second one directly addresses the main test article by a detailed replication. Different defect layouts for size and location are simulated, and strain lines are acquired. Elementary parameters are originally introduced to describe the detected failures, synthetically. A proprietary SHM code is finally applied to check the possibility of using the aforementioned data to detect the presence of the applied imperfections. The outcomes provide hints for the sensor network design, its operational deployment, and acquired information decoding.

## 3. Motivation of Monitoring Systems Assessment

As mentioned above, advantages deriving from the use of bonded solutions for composite structures are reduced by the need to comply with standards, such as the AMC20-29 [21], that report almost integrally what is stated by applicable norms [22] and establish that such recommendations shall be implemented on many airplane categories. In a few words, having identified as critical bonding joints, the ones that could lead to the loss of the whole aircraft, it is affirmed that such connections must be designed in such a way to demonstrate limit load capability by one of the following methods:

1. The maximum disbond of each bonded joint should be consistent with the capability to withstand the prescribed loads, as determined by analysis, test, or both (solution → insert mechanical connections), or
2. Proof testing is conducted on each production article by applying the critical limit design load (solution → massive testing of each part), or
3. Repeatable and reliable non-destructive inspection techniques are established to ensure the strength of each joint (solution → introduction of SHM systems).

The first criterion is the one currently used and is implemented by adding fasteners to prevent the growth of flaws beyond sizes compatible with limit loads bearing; it implies additional weight and a longer operation time. Furthermore, drilling composites may introduce further risks of damage onset, which may be confined by increasing the thickness, and therefore adding further weight. Bonded solutions are also penalized in the case of repair patches: if adhesives are used, the non-repaired structure is required to exhibit the ability to withstand limit loads increased by 20% [23].

The second criterion is almost impracticable, since it would require a devoted test for each released part. In addition, since this arrangement does not allow predicting the degradation of glued joints by chemical and mechanical agents over time, other experiments should be practiced.

The third criterion represents a fascinating option, since it does not require additional mechanics but the continuous monitoring of the state of the joint. Currently, a consolidated and comprehensive tool enabling the assessment of the connection strength does not exist. Novel SHM techniques, based on a better understanding of the physics behind damage, mechanics, and the presence of flaws, could offer new perspectives.

The work presented herein is collocated in a larger activity aimed at setting up an SHM system for the prompt identification of disbonding areas smaller than the critical ones by frequent checks, for instance, occurring after each flight. Both adhesive imperfections after manufacturing and their deterioration during operational life are targeted, intending to bypass the use of additional mechanical joints. In that frame, the study reported in this paper proposes the characterization of the strain function under damaged conditions through relevant parameters and tests the possibility of using that information in a suitable detection algorithm. The adoption of distributed sensing technology is the key element of the adopted damage monitoring strategy.

## 4. Distributed Sensing by Fiber Optics

With an overall diameter of hundreds of microns, optical fibers are minimally intrusive devices [24], with the further capability for hosting several sensors on a single line. Such properties make them particularly suited for integrated SHM applications, which require a huge amount of information to work properly, since a structure has infinite degrees of freedom. The use of embedded layouts is a fascinating way to approach SHM and is technologically possible by means of FO. Several authors have worked on this concept, proposing both passive and active outlines, for instance, aimed at detecting acoustic emission events correlated to crack generation [25], or the profile reflection of Lamb waves generated by piezoceramics, which have proved to be effective on simple or localized regions (the already-mentioned *hotspots*) [26].

Fiber optics have reached maturity for strain and temperature sensing. Many commercial devices are available on the market, including some certified for flight [27]. They are commonly used in many other fields, as in the case of large industrial and transportation infrastructures [28,29]. Excellent reviews on FO technology application on aircraft composite structures may be found in the literature, as in [30]. Fiber optic architectures for structural strain monitoring can be basically distinct in:

1. Point sensors, to detect strains and their variations at specific places along the structure; such a configuration is interesting for applications in so-called *hotspots*, i.e., regions characterized by a high risk of damage onset by excess of mechanical stress [31];
2. Sensor arrays, similar to the above but multiplexed along a fiber, such as a series of fiber Bragg gratings (FBG), usually in a number not exceeding 16; such a layout allows interesting reductions in cabling and related routing [32];
3. Distributed sensor arrays, expanding the potentiality of the former possibility till attaining device density up to several hundred per meter [33,34]; this outline permits us to achieve experimental insights resembling a classical numerical output.

Distributed sensing allows following adhesive effects’ discontinuity along the bonding line, continuously [35]. This is very important if it is considered that such a kind of fault concentrates its effect in a limited region of the structure, so that sensors located even a few cm away do not distinguish any variation with respect to the nominal (integral) configuration [36]. Rayleigh or optical frequency domain reflectometry (OFDR) is a technique for high spatial resolution distributed sensing [37], which is implemented herein. It has been applied to UAV [38] and even to large civil constructions [39]. Other methods with similar capabilities are available, and a synthetic comparison is reported in Table 1 [40]. Only OFDR systems offer the mm resolution suitable for aircraft SHM needs [41].

## 5. Coupon FE Analysis

**Figure 1:**
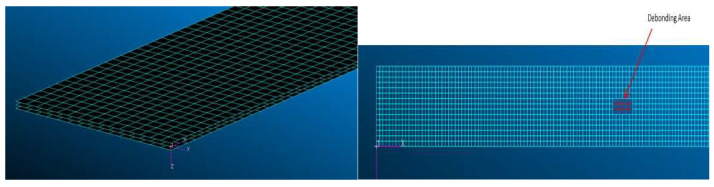
A FE representation of the ASTM Coupon.

With the objective to achieve some preliminary confidence in the strain information type that can be acquired by distributed fiber optics in the presence of adhesive flaws, a preliminary FE analysis was performed on a thin, uniform composite plate. The aim of this first analysis was to obtain some hints on the area of influence of local debonding in terms of strain levels modification and the shape of the deformation function along the bonding line. As a reference, a typical coupon under a three-point bending load was selected, in conformity with the ASTM D709 standard [42]. The 150 × 15 mm coupon was made of two superposed laminates, each 1.5 mm thick, with a stacking sequence ±45\45 3 s, and bonded to each other by a 0.5 mm-thick adhesive layer. The mechanical properties of the single laminae and the adhesive material are reported in Table 2 and Table 3. The FE model implemented 2D QUAD elements to simulate both the cited materials; a 1 mm mesh was adopted, simulating gluing contacts among the interface grids (Figure 1). A de-bonded area was simulated at the middle of the coupon model, 3 by 6 mm wide, as a discontinuity in the Young modulus of the related elements. In this way, the FE continuity was preserved, concentrating the missing adhesive effect only in the stiffness properties. In this first study, the attention is not focused on actual strength and deformation limits of the investigated material, since the activity is exclusively aimed at investigating the strain profile characteristics; on the other hand, linear models were adopted.

The investigation concerns two relevant aspects related to the bonding damage effects characterization: the influence of the load position and the influence of the damage location on the strain output. Acquisition is performed along a straight line, deployed spanwise, and simulates the presence of a distributed optical fiber.

**Figure 2:**
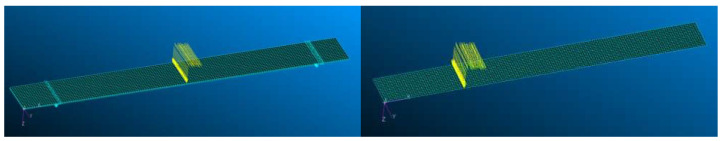
Numerical loads test cases: symmetrical, and non-symmetrical.

**Figure 3:**
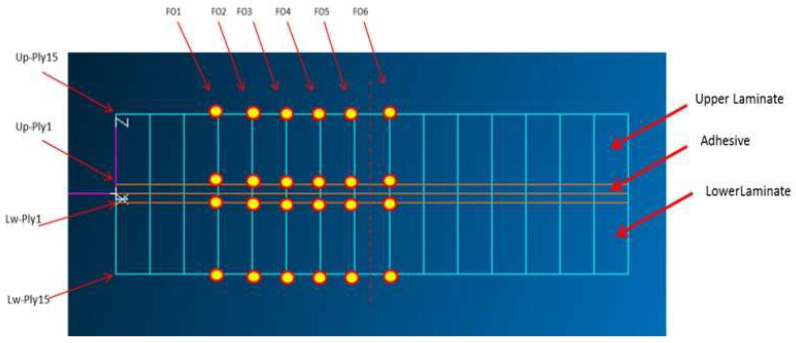
Location of the virtual distributed optical fibers.

External forces are placed at the middle and another arbitrary point, within the inner support region, Figure 2. The results attained on undamaged and damaged coupons are acquired and compared by considering bundles of six parallel virtual fibers, arranged along the longitudinal mesh lines at different thickness levels, Figure 3, for a total of 24 dense arrays.

Several FE analyses are performed for different values of forces in order to check the reliability of the numerical representation. Case N.3 outcomes, referring to a 500 N load applied to each node along the outline shown in Figure 2, are reported for Ply1 (top layer) and Ply15 (bottom layer) of the upper laminate (Figure 3).

**Figure 4:**
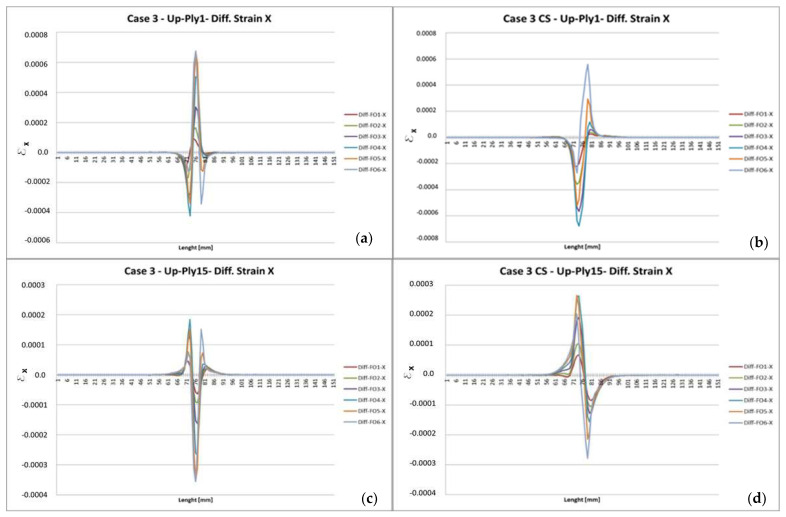
Difference of longitudinal strain on upper laminate: Ply 1 (external), symmetrical (**a**), and non-symmetrical load (**b**); Ply 15 (bonding interface), symmetrical (**c**), and non-symmetrical load (**d**).

**Figure 5:**
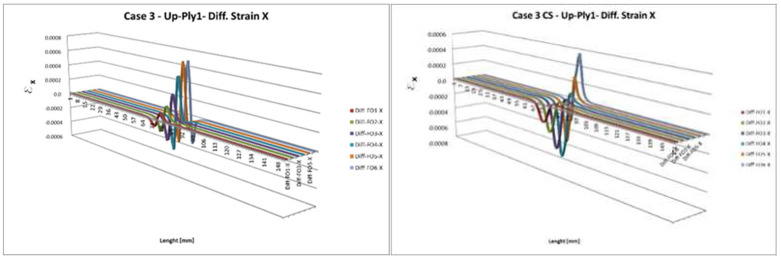
A 3D representation of Figure 4a (**left**), and 4b (**right**).

The individual curves presented in Figure 4 represent the difference in the spanwise strain values acquired in the damaged and undamaged cases. A 3D view (Figure 5) permits a further understanding of the strain difference behavior in the presence of a debonding area. Several considerations follow:

1. The damage effect is sensibly confined within the damage location; in fact, far away from that location, the strain difference between damaged and undamaged conditions approaches zero;
2. As expected, the transversal strain difference is significantly dependent on the distance from the fault location, with a deviation approaching almost one magnitude among the various regions;
3. The load position influences the shape of the deformation difference profiles greatly: the diagram changes from a fully symmetrical to a quasi-non-symmetrical shape, as the external forces are placed at the middle span (symmetrical configuration), or at a lateral station, respectively;
4. Absolute strain variations related to symmetrical and non-symmetrical load outline attain the same magnitude.

These reflections allow highlighting some critical aspects, directly affecting the use of those strain data for SHM purposes:

1. Damage evidence depends largely on the distance position between the optical fiber and the fault;
2. Deviation strain profiles are affected by the load type;
3. In order to increase the extinction area (i.e., the region where the fault effects are clearly visible), measurement accuracy is a critical factor.

As the actual damage size and the extension of the area where strain difference is appreciable are compared for this specific test case, further hints may be guessed:

1. The damage effect extends for almost five times the flaw length;
2. Such an amplification measure seems uncorrelated with respect to the fiber position and the load distribution (Figure 4 and Figure 5);
3. The strain difference profile is centered at the middle of the damage location, with the following peculiarities:
4. For symmetrical loading, the center of damage location coincides with the maximum strain variation;
5. For non-symmetrical loading, the center of damage location corresponds to a zero-crossing.

## 6. Reference Test Article and FE Model

**Figure 6:**
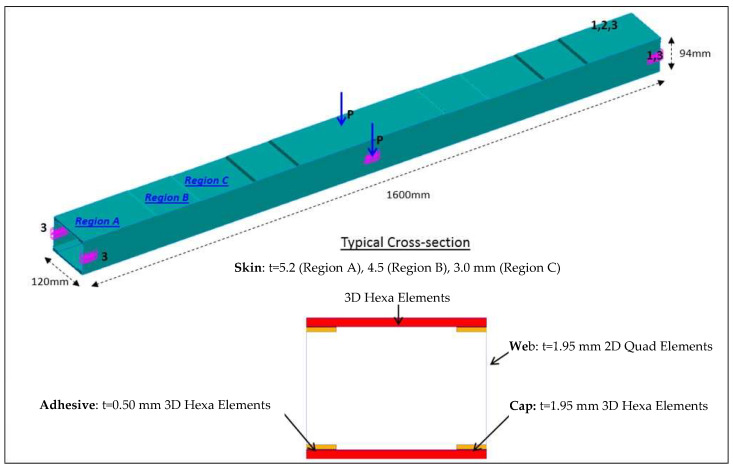
FE representation of the test article design [36].

The study is concentrated over a test article representing a typical aircraft wing element (Figure 6) [36]. It is made of two unidirectional carbon-epoxy plates deployed on its top and bottom, and two C-type symmetrical beams as lateral walls. Such four parts are bonded together using a typical structural paste. The structural components and the adhesive layer are mostly modeled through solid connection elements, HEXA. Three-dimensional equivalent orthotropic materials are then assigned to each of them. Only spar webs are modeled using two-dimensional elements, QUAD. Since the upper skin is compressed under common operations, it is assigned with a variable thickness to enable quasi-uniform stress distribution along the span. Reinforcements are evident at the beam extremities for facilitating loads application in lab.

**Figure 7:**
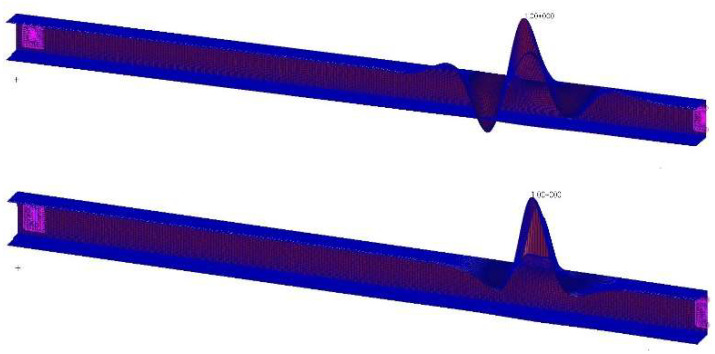
FE buckling analysis.

The specimen was designed by considering the classical safety factor 1.5 with respect to its behavior, as all its elements are defect-free. The maximum allowed damage is then determined by numerical analysis. The structural system is found to lose about 35% of its capability of bearing loads as a debonding of 100 mm magnitude is imposed to its top caps, therefore moving its robustness at the acceptance boundary (safety factor reduced to about 1). Buckling studies give evidence of this circumstance: while the first buckling eigenvector does not change, qualitatively, the maximum allowable force decreases from 8.0 to 5.2 kN (Figure 7). Therefore, it can be stated that a critical debonding size, i.e., intended as the one capable of canceling the protection insured by the safety factor, is around 10 cm.

## 7. Test Article FE Analysis

Numerical analysis of the test article was performed by considering two load types:

1. Symmetric load;
2. Generic loads.

**Figure 8:**
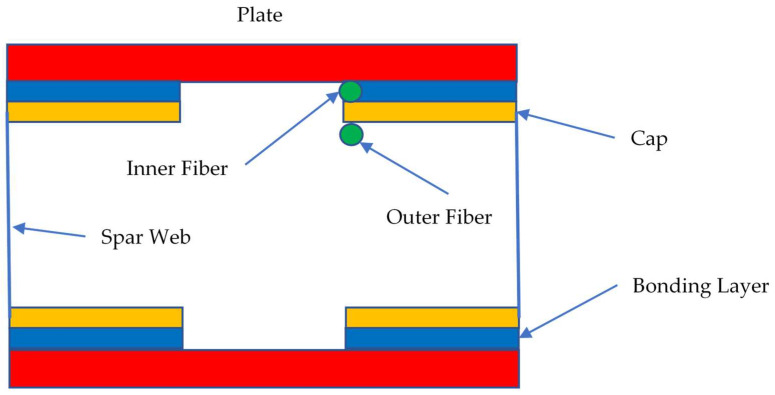
Pictorial view of the composite beam section; dimensions not to scale. Plane location of the strain acquisition lines, simulating a virtual, distributed fiber.

In the first case, two 2 kN forces are deployed on each sidewall of the reference composite beam. In the second one, the same concentrated forces are positioned at a generic station of the test article, plus a slight difference between the loads to insert a torsion component and make the excitation system more generic. Acquisition lines are located at the external and internal caps of the C-beam (Figure 8). In detail, the inner location is directly in contact with the adhesive layer, while the outer location corresponds to the inner open side of the beam section. In the numerical investigations, these defect types are applied:

1. Non-symmetric (cap-partial);
2. Non-symmetric (cap-extended);
3. Size: 10/20 mm per 10/120 mm.

In the list above, non-symmetric means that the damage is located on a generic section of the structure, not belonging to the transversal section equidistant from the supports, so as to approach a realistic situation. Cap-extended and cap-partial refer to the possibility of having the adhesive interruption running all over the cap width or just its half, respectively. Size is referred to a cap-wise per spanwise extension.

**Figure 9:**
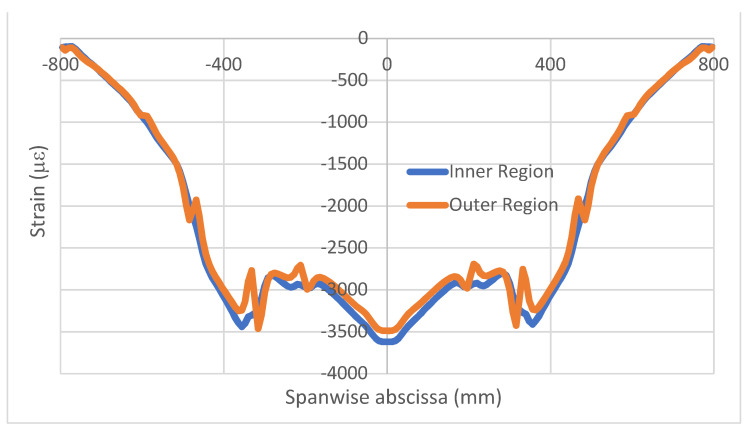
Strain profiles, inner (**bottom**) and outer (**top**) cap fiber; symmetrical load.

**Figure 10:**
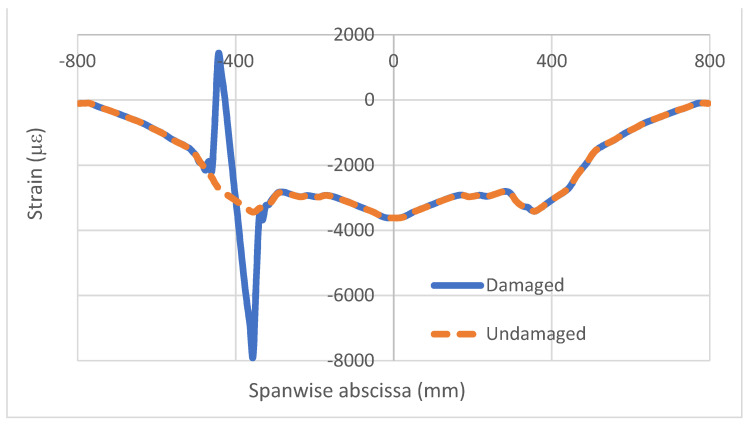
Strain profiles for locally de-bonded spar at the inner cap fiber.

**Figure 11:**
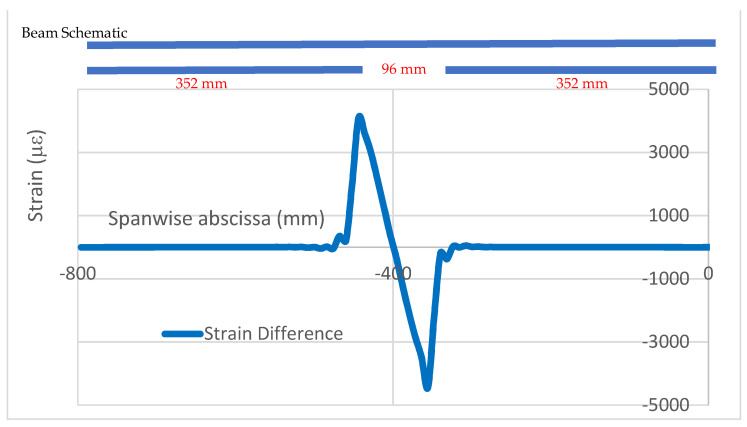
De-bonding effect and its localization (applied damage extension: 96 mm).

An example of the strain analysis for a damaged and intact bonding layer is now reported, referring to symmetrical flexural excitation (load type N.1). In that case, a 96 mm-long debonding, displaced along the whole cap, is considered, placed at 352 mm from the free end of the beam. The strain profiles represent the longitudinal strain, acquired in the longitudinal direction, namely ε_x_(x). In that example, thickness irregularities affect the deformation output and are much more evident in the external acquisition line (Figure 9) relative to the undamaged structure. According to this result, it is decided to use internal fiber data as the main reference, favoring signal cleanness. As expected, the introduced flaw, sized 96 × 20 mm, causes a sound difference in the damaged region with respect to what was achieved in the case of an integral structure only in the proximity of the affected zone, coherently with the outcomes of the preliminary tests on simple coupons (Figure 10). The effect localization is so evident that the rest of the structure, particularly the homogeneous region, may be taken as a reference for further observations. In other words, symmetrical parts under symmetrical loads show the same structural response, irrespective of the presence or absence of local irregularities (adhesive discontinuity). By operating a simple difference between the values of the two curves, a new graph is obtained and reported for only the left half of the beam (Figure 11). The resultant curve has evident antisymmetric characteristics. The small oscillations appearing at the start of the curve seem related to the adopted mesh step, equal to 8 mm, and are therefore much larger than the one used in the preliminary investigations on the simple coupon (1 mm). At the top of the picture, a schematic of the spar caps is reported, showing the location of the imposed damage as a forced interruption of the bonding continuity.

These preliminary results highlight and confirm some outcomes derived from the preliminary study of a simple coupon. In particular, for the investigated case (about 100 mm flaw), the following are confirmed:

1. The local characteristic of the damage effect, in turn implying:
  - The undamaged structure may be used as a reference, since the variation of its response is limited to the damage zone;
  - For a symmetrical structure under symmetrical load, symmetrical parts could be used as reference too;
2. The excess of the affected area with respect to the flaw size;
3. The effect in terms of strain variation is relevant, i.e., the strain variation amplitude is the same magnitude than the original structural response.

## 8. Numerical Elaborations—Symmetrical Load

**Figure 12:**
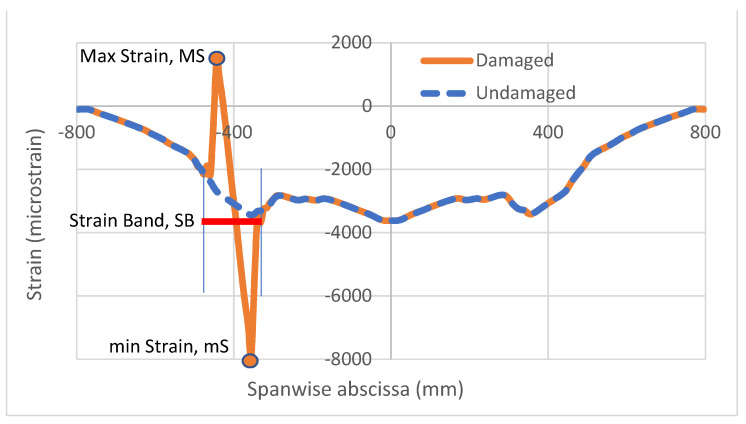
Strain deviation parameters, [36].

After these first computations and these preliminary considerations, a limited number of parameters were defined to try to assess a synthetical representation of the damage effect on the strain function. These are introduced in Figure 12 [36] and are described as follows:

1. Maximum strain, MS, and minimum strain, mS, intended as the relative maximum or minimum values attained by the deformation as a consequence of the flaw effects on the structural response;
2. Strain band, SB, defined as the points at which the function irregularities begin; in spite of the pictorial evidence, it is difficult to come to a rigorous definition of this parameter, which would imply a sort of tolerance after which the deviation starts to be appreciable, and this, in turn, could be dependent on the specific problem.

**Figure 13:**
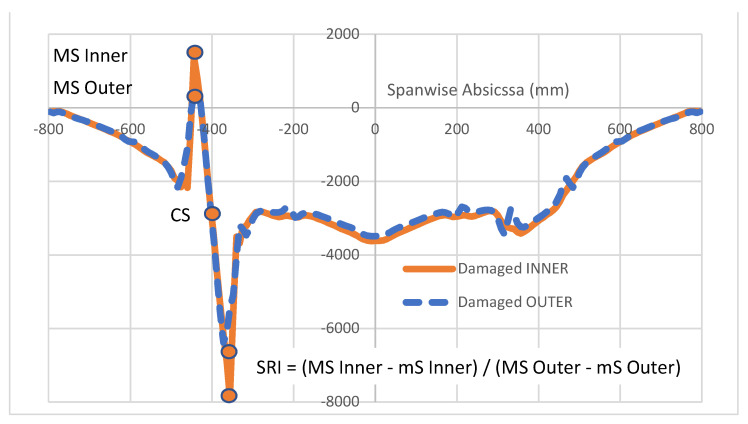
Strain variation (µε) characteristic parameter, related to arrays differently deployed.

Another parameter that may exhibit some importance is the Strain Ratio Index, SRI, defined as a ratio of a combination of the reference MS and mS, as defined above but in a different domain, vs. the same combination of measures referring to other sensors deployed in the same area of interest (for instance, the fiber deployed outside the cap and recalled in Figure 8). Its meaning is illustrated in Figure 13 and may be expressed by the following relation: SRI = (MS − mS)_ref_/(MS − mS)_gen_. Even though such a quantity may have its significance in further analysis, it was abandoned in the current study.

After these considerations, further numerical analyses are carried out for different debonding lengths, leading us to compile the following table, Table 4, reporting the resulting values of the characteristic parameters. In the table, it is preferred to insert the difference between the maximum and minimum values of the strain in the damaged region, MS and mS, instead of their absolute values, having considered such a difference mostly significant in the description of the phenomenon (delta strain, DS = MS − mS). An alternative possibility would be to introduce the sum of the absolute difference between each of the strain peak measures and the strain center-bandwidth value (CS in Figure 13). This option would lead to the same result in the referred case but could have significance in dealing with peaks of the same sign.

In these outcomes, it should also be taken into account that the estimation reflects the adopted mesh step (8 mm), which has inevitably conditioned the data resolution. Furthermore, it should be recalled that, in the strain band, SB, the evaluation is influenced by the already-cited value oscillations in the vicinity of the strain variation onset. Another remark concerns the type of debonding that was considered in this evaluation: in order to avoid any boundary effect, it is supposed the flaws are always extended though the entire cap width. In this way, the optical fiber always passes through the damaged region.

At this point, two combinations of the above parameters are also introduced. It seems reasonable, for instance, to normalize the SB with respect to the damaged length, thus obtaining a non-dimensional value (normalized band, NB). In the same way, it is interesting to insert the ratio between DS and the debonding extension so as to evaluate the tendency of the strain difference for larger and larger debonding regions (normalized delta, ND). A further table can then be proposed (Table 5).

**Figure 14:**
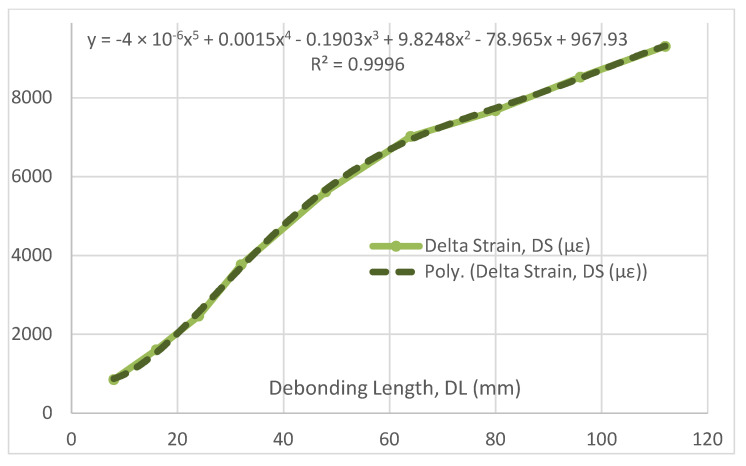
Delta strain as a function of the debonding length, and its polynomial approximation.

**Figure 15:**
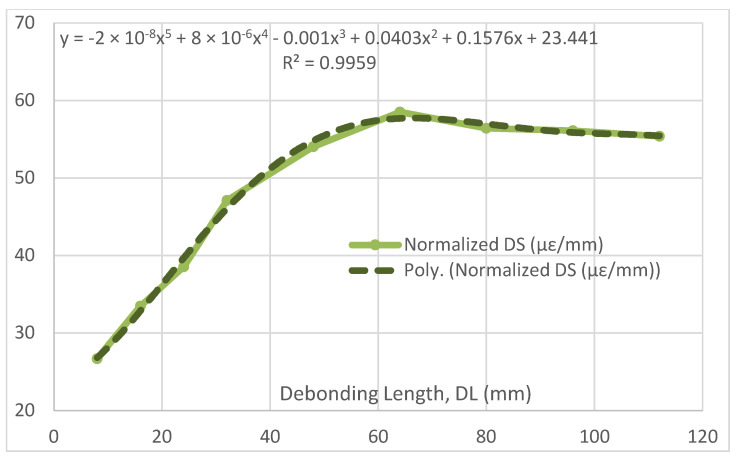
Normalized delta strain as a function of the debonding length, and its polynomial approximation.

Such values may be plotted on graphs to evidence a possible functional dependence of the different parameters on the damage length. First, DS vs. DL is reported in Figure 14, while its normalized version is shown in Figure 15. The ratio function evidences the variation in the functional relationship between the two parameters from the linearity (in a perfect linear correlation, the result should be a constant). In the former picture, three distinct regions may be evidenced: the first and the second ones seem to be an expression of a different potential relationship, connected through an inflection point, while the last one seems closer to a linear relationship (i.e., the strain difference grows in the same measure than the flaw dimension). It can be remarked that a non-trivial polynomial approximation (5th degree) approaches the curve extremely well, leaving this point open for further investigations. The latter picture confirms the three-region aspect before being individuated, as well as the emerging linear relationship between DL and DS for higher values of the debonding length. Furthermore, the linear appearance in the first segment (or first region) would indicate the possibility of a squared dependence between DL and DS for small values of DL (in the graph, less than 40 mm). Finally, it is observed that, in view of further analyses extended to other structures, these results should also be assumed to be valid for the investigated case and the related test article; different geometries and complexities could lead to additional considerations, even more diverse from the one reported here.

**Figure 16:**
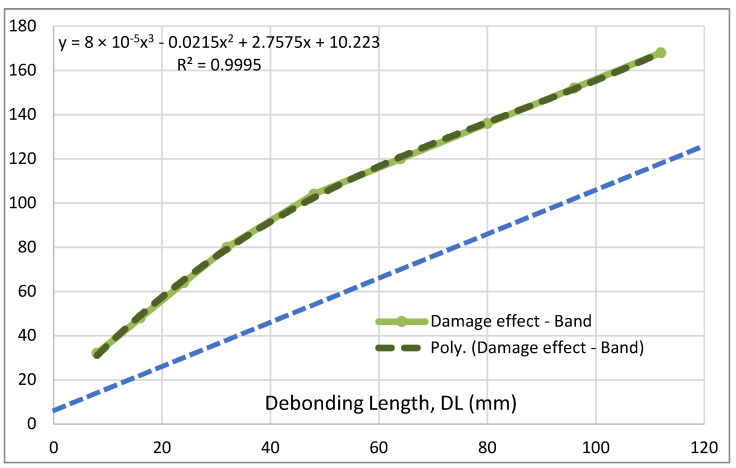
Damage effect width vs. actual debonding length; in blue, the reference linear link (1:1).

**Figure 17:**
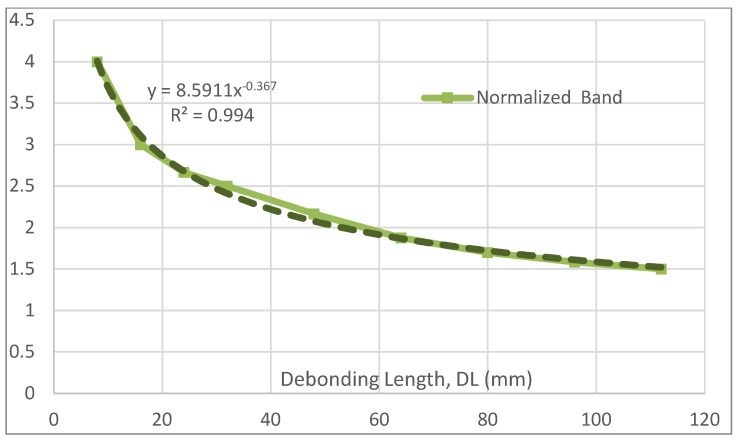
Normalized, non-dimensional damage effect width vs. actual debonding length.

Moving to the other parameters, a representation of the debonding region effect is illustrated in Figure 16. As can be seen, such a behavior is characterized by two distinct regions, both almost linear. The first one, in the approximated range DL [10; 40], shows that the area whose structural response is influenced by the flaw presence is larger than the flaw itself. In that case, it can be shown the first segment of the curve is parallel to a straight line with an angular coefficient equal to about 2 and is shifted towards the top by an amount of about 20 mm. This, in turn, means that the ratio among the affected region extension and the bonding irregularity size would grow as this latter value approaches zero. Of course, it is expected that a further investigation in that area (i.e., for extremely small values of the damage) would show a further curve segment joining the origin with the curve. In the second zone, approximatively in the interval DL [50; 120], the relationship between the effect and the flaw appears to be parallel to a line with angular coefficient equal to 1 and shifted towards the top by about 60 mm. This straight line is indicated with traits in Figure 16. A small fitting region in the approximated range DL [40; 50] completes the functional representation. This curve is very well approximated by a cubic polynomial, whose expression is reported in the graph. By applying the same criterion as before, the strain band is normalized (made dimensionless, in this case) by being divided by DL to identify possible linearities. However, in spite of the mentioned evidence, and due to the shifts above recalled, the resulting curve does not show any constant segment. Instead, it is well-approximated by a power interpolation, as reported in Figure 17. The exponent is, by chance, very close to (−1/e), encouraging further studies into smaller and wider flaws applied to models with finer meshes.

## 9. Non-Symmetrical Loads and Other Damage Configurations

**Figure 18:**
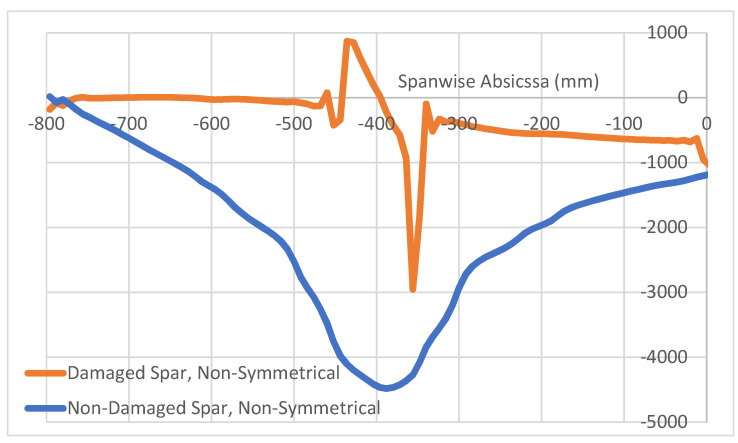
Non-symmetrical load: longitudinal strain function for damaged and non-damaged spar.

This result holds only for symmetrical loads on a symmetrical structure. As a non-symmetrical load of the same resultant magnitude is considered, over the same damaged structure, something different happens. The situation is reported in Figure 18, where the spanwise longitudinal strain along the left and right top caps are reported, referring to sensor fibers deployed within the bonding layer between the C-beam top cap and the top plate. As before, the non-damaged cap exhibits a regular spanwise deformation function, while strong irregularities appear in the fault-bearing cap. In this case, however, the strain function is considerably different from the previous sketches, showing two marked peaks in the extremal region of the flaw.

**Figure 19:**
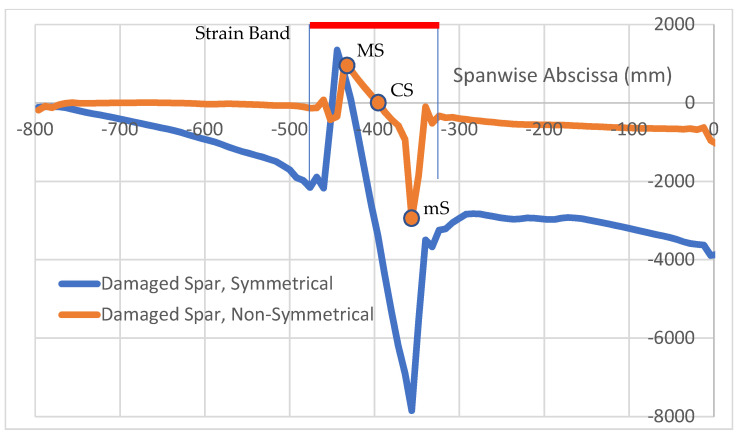
Non-symmetrical vs. symmetrical load effects: longitudinal strain function for damaged and non-damaged spar.

It may be interesting to compare the strain profiles derived from symmetrical and non-symmetrical loads to verify if some commonalities still hold. As reported in Figure 19, the so-called strain band (SB) may be identified even in this case, and it does appear to have the same extension as in the previous case, but with a very slight shift on the left side of the picture and equivalent to 8 mm, the mesh step. Therefore, it can be easily attributed to a numerical error. Equally, even the strain peaks are easily identified and may be associated with the values of maximum and minimum strain, MS and mS. Another common point is the presence of a straight line in the middle of the discontinuity to indicate a linear characteristic of the function ε_x_(x) in that range. Some of the main differences are listed here. The straight lines just mentioned do not indicate MS or ms directly but interrupt to a certain moment, exhibiting a strong discontinuity just before the peak is reached. This event occurs differently at the flaw effect start and finish sides. The magnitude of the difference of those peaks is strongly reduced (4000 vs. 10,000 µε); it should be, however, considered that, in this latter case, strain levels on the damaged spar are significantly lower (maximum nominal level is under 1000 µε, vs. about 4000 µε than in the previous case). An analysis of the strain function characteristic parameters was not performed in this case, postponing dedicated investigations to future studies.

**Figure 20:**
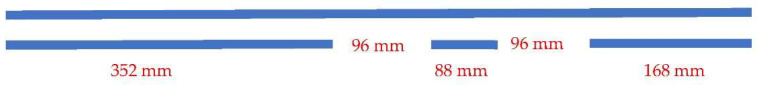
Schematic representation of the beam under investigation, half span. Visualization of the top caps of the longitudinal C-spar. Position of the flaws.

**Figure 21:**
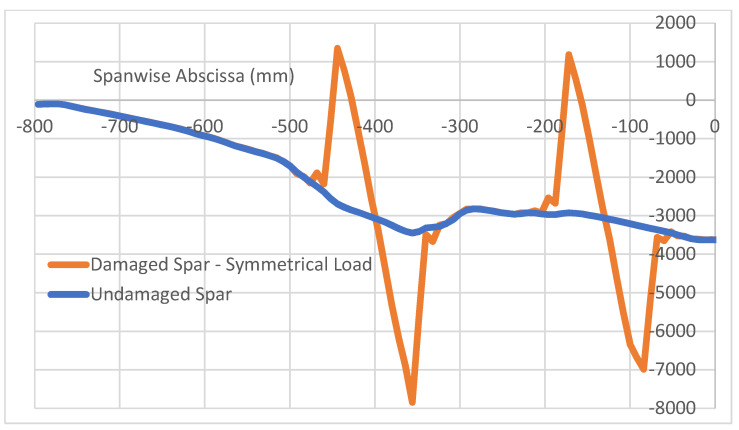
Effects of a 2-flaw array on the spar longitudinal deformation (µε); symmetrical load.

**Figure 22:**
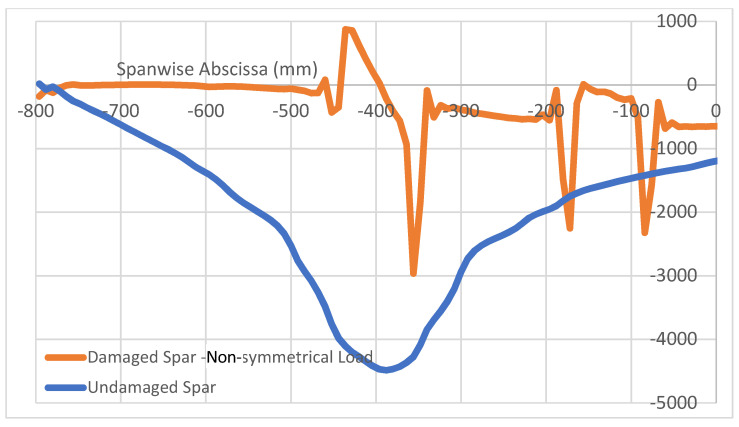
Effects of a 2-flaw array on the spar longitudinal deformation (µε); non-symmetrical load.

**Figure 23:**
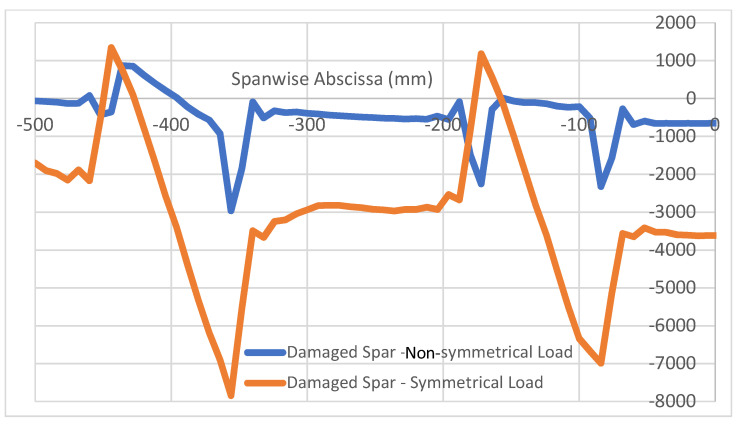
Comparison of damage array effects on deformation (µε) at two different stations deployed along the span of the beam. Reported spars are the ones where the flaws are deployed.

The above results show the characteristic localization of the damage effect, confined within a small area around the flaw itself. In fact, in the analyzed case, as debonding is placed in a certain area of the top cap of a spar, the other regions behave in the same way as for the intact structure; in particular, the structural response of the unaffected (integral) spar is equal in the two cases. It should thus be expected that multiple damage insurgences should give distinct effects in each one, and that the properties that are identified for the occurrence of a single flaw are also found if many discontinuities are deployed. In other words, flaws should not interfere with each other unless the flaws themselves merge in a larger irregularity or, more precisely, until the affected regions (as seen, larger than the nominal defects’ extension) start to overlap. A numerical test is then performed, introducing a couple of flaws at a small distance from each other on the same spar, and making the structure undergo the same load history than before in the symmetrical and non-symmetrical configuration. In detail, two debonding areas sized 96 mm and 88 mm distanced from each other are applied, as briefly schematized in Figure 20. The reported curves in Figure 21 and Figure 22 are relative to the longitudinal strain data acquired along both the spar caps within the bonding layer, as in the previous cases. The damage effects are clearly separated in both the symmetrical and non-symmetrical load configurations. It is evident that the same characteristics exhibited and defined before still hold and maintain the same values as before. For the symmetrical load, the slight variation between the two damage effects is related to other beam characteristics such as local thickness, the different basic deformation reference, and so on (Figure 21). No apparent interaction is detectable. The same outcomes derive from the application of the non-symmetrical load, as previously defined (Figure 22). In this latter case, a larger difference in the deformation diagram shapes may be observed between the one at a quarter span and the other towards the center. Such a behavior could be an index of the effect of general loads on the structural response of damaged components, which could indicate that the flaw effects may significantly vary in the dependence of the specific region where it is insurgent, for the same load and the same damage level. The geometrical variation particularly influences the strain peaks that are much closer in value and exhibit the same sign (deformation reduction). In this case, the use of alternative descriptors involving the middle strain value, as previously introduced, could have some applicability. For the sake of completeness, the strains related to damaged curves are jointly reported in Figure 23. The length of the regions that look affected by the damage presence do not macroscopically change as the kind of load varies, even if the shape of the strain function is very different.

**Figure 24:**
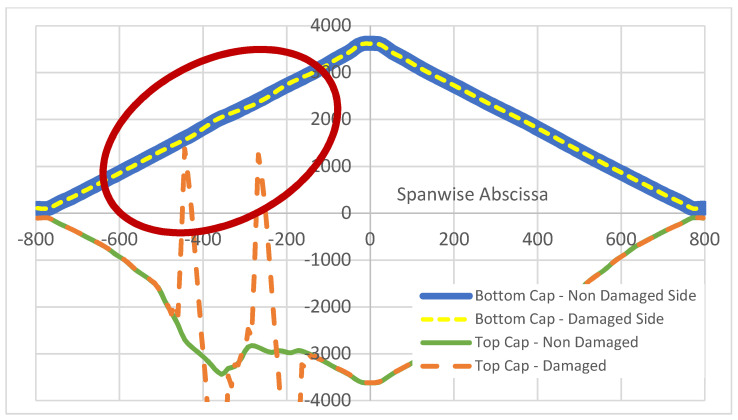
A comparison of the longitudinal strain functions in the bottom and top caps of the two C-spars; evidenced in the red circle, a slight modulation of the signal revealed in the bottom cap of the damaged spar.

**Figure 25:**
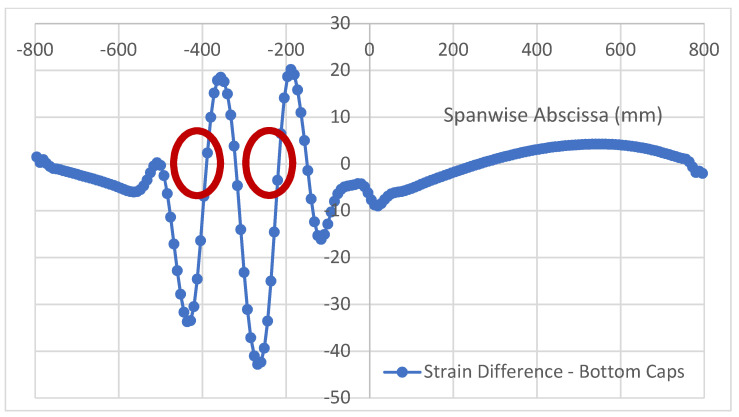
Strain difference function (µε) between left and right bottom caps, retrieved within the bonding line. Red circles indicate approximate positions of the applied debonding at the top cap.

Top and bottom caps of the referenced structure show significant differences in their behavior, since the latter has a uniform thickness. Therefore, under a bending load caused by a point force acting at the mid span, a classical linear diagram of the deformations arises. Moreover, the two bottom caps, confirming the very localized nature of the damage, do not exhibit any macroscopic difference, at the point that it is possible to state that the function representations are superposed. In Figure 24, the longitudinal strain functions for the bottom caps are reported, together with the deformations diagram relative to the already discussed non-damaged top cap, as a reference. In the picture, within the red ellipse, it is, however, possible to see a smooth oscillation of the strain concerned with the bottom cap of the same spar, where disbonding was applied. As this is zoomed in, after having operated the usual strain difference between the left and right bottom caps, a clear variance in the aspect and magnitude of the strain function shape is revealed (Figure 25). Such deviations assume the shape of an almost regular, modulated sinusoidal sweep oscillation. In detail, the curves are well-shaped, and the point density is equally distributed between the growing and decreasing segments of the path. It is worth noting that the effects of the flaws seem to merge in a single region this time and produce a single blended, smooth curve. The absolute magnitude of the strain difference is two magnitudes smaller than the previous case; the estimated tens of microstrains are at the limits of standard measurement systems’ threshold for real applications, where the background noise is produced at the same level. In spite of the speculative interest of the detected phenomenon, this latest consideration would indicate the hard applicability of the related outcomes.

**Figure 26:**
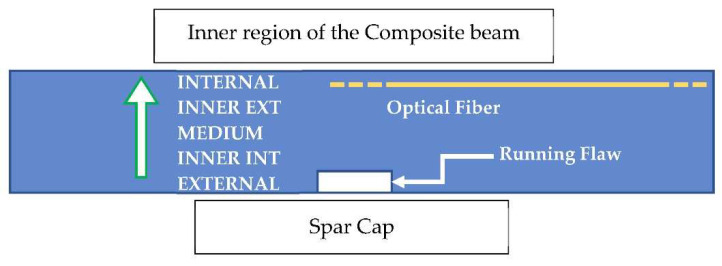
Schematic representation of the imposed disbonding, and the codification of the N.5 subsequent positions along the cap. View from above; internal section of the composite beam deployed on the top of the picture. This schematic is the reference for the data shown in all the other pictures (Figure 27, Figure 28, Figure 29, Figure 30 and Figure 31).

**Figure 27:**
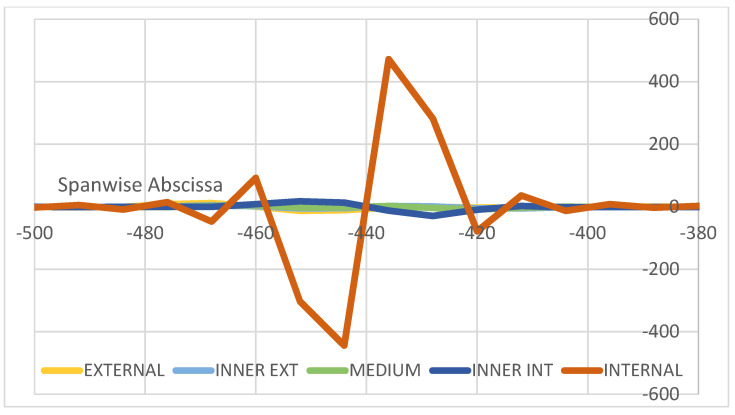
Strain difference, consequent local adhesive imperfections placed at different stations along the C-spar top cap, as measured by an optical fiber deployed at the inner part of the cap itself, within the bonding layer.

**Figure 28:**
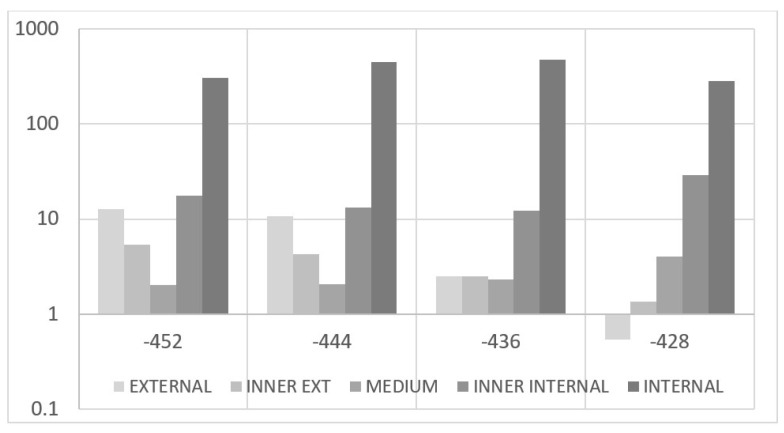
Histogram representation, in a logarithmic scale, of the strain difference, measured by the same optical fiber as the flaw is moved from one side to the other of the cap. Reported values refer to four different abscissas, spanwise, in the region of the max difference (internal fiber, Figure 26).

These preliminary investigations on the reference test article make it possible to state the strong localization characteristics of the debonding defects. It is almost consequential, then, to explore the effects of a flaw insisting on a partial spanwise region of the cap, i.e., non-extended over the entire cap width. This analysis is devoted to verifying the actual possibility of observing the presence of debonding by means of a dense sensor array that may or may not cross the flaw area. In this case, a very small adhesive imperfection is implemented at the limits of the numerical mesh discretization, sized 16 × 8 mm spanwise vs. cap-wise. As for the other analyses, the optical fiber is simulated within the bonding layer of the C-spar, towards the interior of the composite beam section. The irregularity was moved along the cap width in five different positions so as to cover its entire width, moving from the external to the internal part (Figure 26). The results are shown below (Figure 27), where the only deformation difference is reported between the acquisition performed on the damaged and undamaged spars, i.e., the spar with or without debonding. Additionally, the graph is zoomed in the zone of the damage; as expected, the other regions show equal values of the strain, giving rise to overlapped curves. As the fiber crosses the disbonded region, strain deviations attain the interesting value of 400 microstrains, which is extremely relevant if the very limited extension of the discontinuity is taken into account. However, as the irregularity is moved towards the external direction, and therefore far from the virtual fiber, the deformation difference falls by almost two magnitudes, to a few tens of microstrains. The magnitude stabilizes for the other positions: its amplitude reduces a bit, till arriving at about 10 microstrains. The sign is not stable and may turn into positive or negative values. If verified, this evidence brings interesting consequences: a flaw shows good probability to be detected only if the strain is acquired in the region where it is present. These considerations combine with what was previously reported about the tests performed on coupons and deserves more attention in further studies. For the sake of completeness, the absolute values of the strains are reported in a logarithmic-scaled histogram for four different stations along the span (Figure 28).

**Figure 29:**
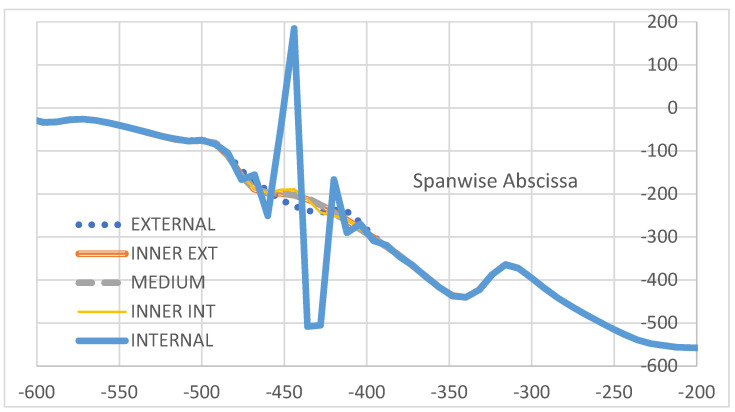
Strain values in the region of local adhesive imperfections placed at different stations along a C-spar top cap, as measured by optical fibers deployed at the inner part of the cap itself, within the bonding layer.

**Figure 30:**
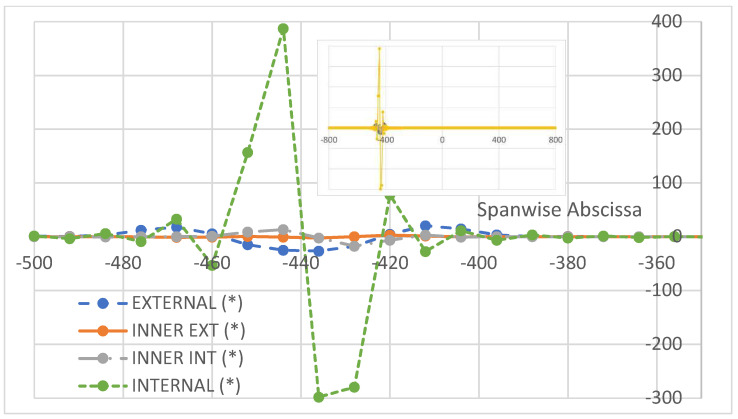
Strain difference between the signal taken by the optical fiber for a certain position of the flaw, and the deformation resulting when the imperfection is placed at the middle of the cap. Focus in the region of the damage. In the small picture embedded, there is an enlarged image of this referenced signal. Values are referenced to those recorded in the case of a flaw placed at the middle of the cap (*).

**Figure 31:**
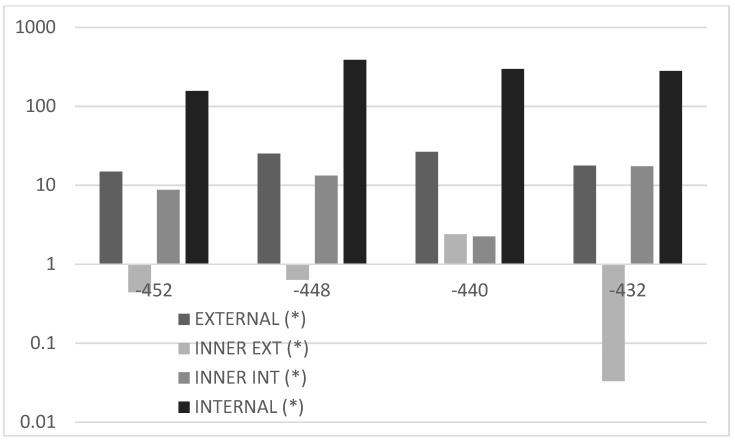
Histogram representation, in a logarithmic scale, of the strain difference, measured by the same optical fiber, as the flaw is moved from a side to the other of the cap. Reported values refer to four different abscissas, spanwise, in the region of the max difference (internal fiber, Figure 26). Values are referenced to those recorded in the case of a flaw placed at the middle of the cap (*).

As a last investigation, the strain difference is evaluated with respect to the action of a non-symmetrical load. In this case, for the non-symmetry of the structural response, the difference between the deformation values recorded along the two spar cannot be implemented. Therefore, the actual values of the deformations are reported. In Figure 29, a zoom on the region interested by the flaw is shown. The shift of deformation amplitude may be evaluated in 400 µε, in line with the previous results. Additionally, the strain information rapidly decays as the acquisition line moves away from the location of the damage and assess its value in tens of microstrains. In order to verify whether it is possible to find a reference for this new set of data, it is chosen to take the medium region deformations, which give rise to the minimum output (Figure 28). The result is reported in Figure 30, confirming the same outcomes as in the previous picture, but for the normalization of the data that drop to zero everywhere else other than in the fault area (enlargement in Figure 30). Finally, a logarithmic-scaled histogram of the values in the zone of maximum strain difference values is reported (Figure 31).

## 10. Numerical–Experimental Correlation

**Figure 32:**
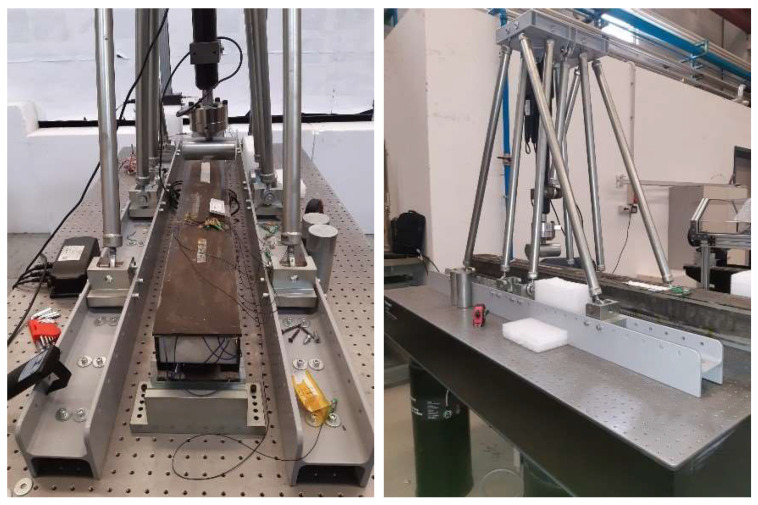
Test rig with the composite beams installed: front (**left**), and lateral (**right**) view.

Experimental investigations were carried out on the composite beam introduced above. A specific test rig was implemented (Figure 32), made of steel tie rods and connected to a Newport Production self-leveling optical table via two I-section steel beams. The optical table was equipped with a network of M6 holes, 25 mm stepped, to anchor the different parts. At the top of the tie rods, a reinforced base hosting a HIWIN LAI-2 linear actuator was mounted. The actuator had a 30 cm stroke and was able to provide a maximum of a 12 kN load. The beam was supported at its extremities by two steel elements, so that a classical three-point bending excitation could be realized.

**Figure 33:**
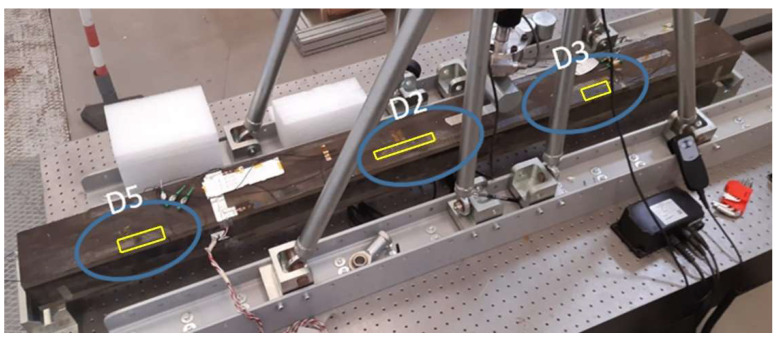
The composite beam with the indication of the implemented flaws.

**Figure 34:**
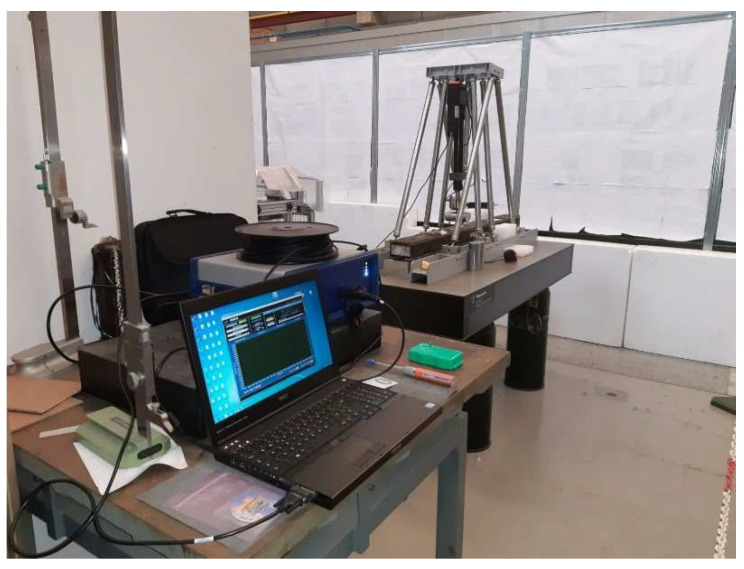
An overview of the experimental set-up: acquisition PC, optical interrogator, optical table, test rig, test article.

A debonding region size that is of interest for aeronautic applications is in the range of the so-called critical length., i.e., the one that makes the safety factor reduce to 1. Of course, it depends a lot on the specific structural system and the operational loads. As the critical length for the reference structure is verified to be in the range of 100 mm, the imposed damage size is chosen to be in that magnitude and is particularly set between 40 and 80 mm, conservatively. The beam is manufactured with some artificial adhesive layer flaws at the interface between the top cap of the C-spar located at the right side of the beam (Figure 32), the left side, and the top plate, with the characteristics reported in Table 6. Such flaws are obtained by interposing some Teflon patches in the corresponding regions during the curing process in order to avoid any physical connection between the aforementioned structural elements. Those patches were deployed in some nominal zones. After the manufacturing, an NDI was carried out to determine their actual extension and positions, so to have a solid data background to set up consistent FE models and to be used for comparing the SHM algorithm predictions. The flaw identifiers are shown in Figure 33; since the original yellow marks (originally used as references during the experiments) are barely visible in the picture, yellow contours have been added *ex post* for increased clarity. D2 discontinuity was deployed at the inner segment of the cap, with respect to the beam section. A distributed optical fiber was placed in the nominal bonding line between the C-spar top cap and the top plate, 8 mm from the external edge. Strain information was processed via the Luna Odisi-B optical interrogator. A complete view of the experimental set-up is shown in Figure 34. During the tests, two different kinds of load were applied:

1. Symmetrical load: a perpendicular force placed at the center of the beam axis;
2. Non-symmetrical load: a perpendicular force placed between the axis center and the supports.

The nominal symmetrical load was slightly shifted from the center line between the two supports so that it did not interfere with the damage D2. The test rig had tools that allowed us to apply different kinds of load on the beam, ranging from a nominal concentrated load (applied along few mm of the beam spam to several centimeters), which permitted us to verify possible effects of the load type on the evidence of the damage effects. Such a configuration was applied for both the symmetrical and non-symmetrical load. Therefore, 0, 50, 88, and 190 mm distributed loads were applied, 0 indicating a nominal concentrated force.

**Figure 35:**
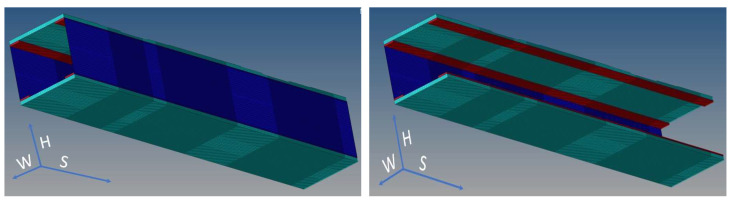
FEM model of the composite beam, with its reference system: H = height, W = width, S = span; integral view (**left**), and view without a lateral wall (C-spar web, (**right**)) to show the C-spar caps for all their length.

**Figure 36:**
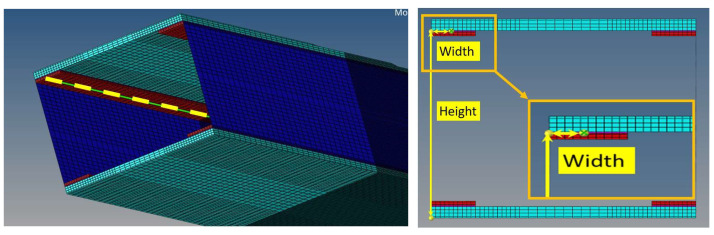
Position of digital optical fiber (**left**) and reference coordinates (height, width) (**right**).

The original FEM model was then adjusted to consider the damage presence (Figure 35). In particular, a finer mesh, 1 mm step vs. the original 8 mm step, was realized in the regions where the faults were present. In order to avoid increasing the complexity of the model too much, such refinement was limited to an extension equal to 5 times the length of the considered fault. The strain values from a row of nodes positioned at the cap interface with the bonding layer were extracted from the FEM analysis and directly compared to the deformation data obtained by the distributed fiber optics (Figure 36). In that picture, the beam section is shown from its back, so that the strain acquisition line appears on the left, exclusively for clarity of representation.

As the damage is expected to cause, after the numerical analysis, a sort of single-wave strain modulation, and referring to the length of the smallest flaw, it is easy to define the minimum step of the sensor array in 10 mm (roughly halving the minimum required length by the Shannon’s theorem, aiming at better addressing the amplitude of the single wave rather than its period, simply). However, the same analyses indicate a sharp behavior of that curve, with the following risk of losing its peak value for coarse meshes. It was then chosen to further reduce that step to 2 mm, which led us to consider at least 10 points (till 11) in the smallest damage area. Furthermore, those same FE simulations show good coherence between 1 mm and 2 mm meshes for the specified debonding regions (in other words, the strain peaks maintain their appearance in the numerical simulations for the two different meshes). Strain sensor arrays that match those characteristics and are small enough to be inserted within the bonding layer were optical fibers capable of so-called distributed sensing and were long enough to cover the whole length of the considered beam for appropriate analyses. Having had some former experiences with LUNA instrumentation (ODISI-B), it was decided to maintain that choice even in this case, for practical and economic reasons, after the technical requirements were met. In detail, the optical fibers used in the experimental campaign were flexible low bend-loss fiber strain sensors, polyimide-coated, with a fiberglass diameter of 155 µm and a minimum bend radius of 10 mm.

Numerical–experimental correlation between the attained data is reported in the following pictures for symmetric and non-symmetric loads. An excellent correlation is found, with particular reference to the damage effect, exhibiting the predicted peaked single-wave shape. Even though the curves do not have a perfect overlap, the proximity remains within the field of engineering acceptability. With respect to the concentrated load, it is evident as the strain output exhibits a singularity for the sharp variation of the correlated values. This phenomenon is confirmed by several observations: a certain distance should be allowed to properly compare the output.

**Figure 37:**
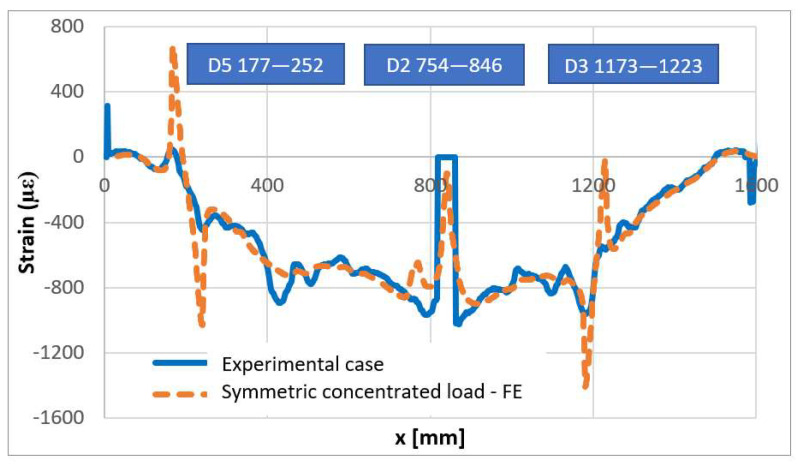
Numerical–experimental correlation—symmetric load.

Moving in the order, the comparison between experimental and numerical curves for the nominal symmetric load is illustrated in Figure 37. The damage effect connected to the flaws D5 and D3 are well individuated, with the major remark that the peak is highly sharper in the numerical simulation than in the experiments. This can essentially be due to the way the fiber is embodied within the bonding layer, so that the deformation step is made more regular by the transmission from the adhesive to its inner core. In fact, the simulation provides information on the theoretical strain that could be read on the structural surface at the interface with the fiber, while in reality, it could be expected for a more complex transmission to occur. This consideration would suggest a deeper investigation into the modeling of the strain transmission between the adhesive and the fiber system, intended as the fiber core, its complex casing, and the surrounding bonding layer. The absence of indications concerning the D2 flaw may be easily associated with the fact that the load is very close to that anomaly, so that it masks the weaker effects of the damage. The numerical response shows a peak in the region of the load application and a close smaller peak, which could be an early index of the presence of the fault.

**Figure 38:**
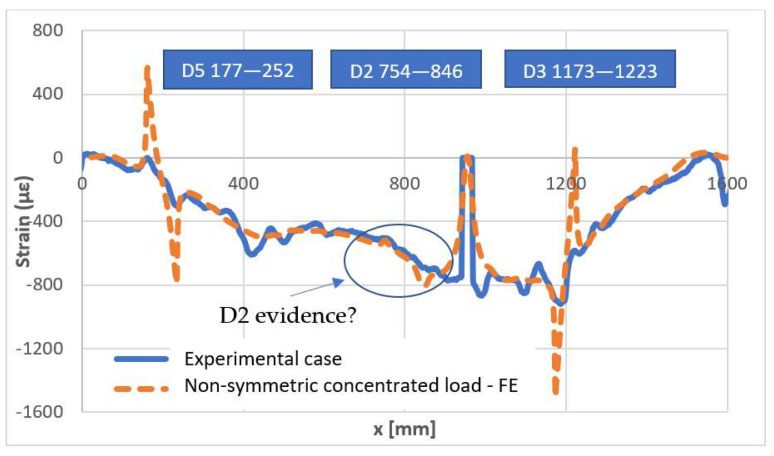
Numerical–experimental correlation—non-symmetric load.

All these considerations hold for the evidence arisen by the application of a concentrated non-symmetric load, Figure 38. The same effects of the damage D5 and D3 may be observed, as well as the same singularity exhibited by the optical fiber in the proximity of the force application. As it is normal, the numerical data provide a smoother strain profile. However, as the load is moved aside, some irregularity in the curve does appear, which could be attributed to the presence of the D2 flaw. Numerical and experimental shapes are very close, in that region, with the numerical data, once again exhibiting a more regular curve. It should be remarked that this feeble irregularity may be connected to the position of the optical fiber that was deployed at about 2 mm from the damage area, cap-wise, thus confirming the forecast abatement of the fault effects beyond its region.

**Figure 39:**
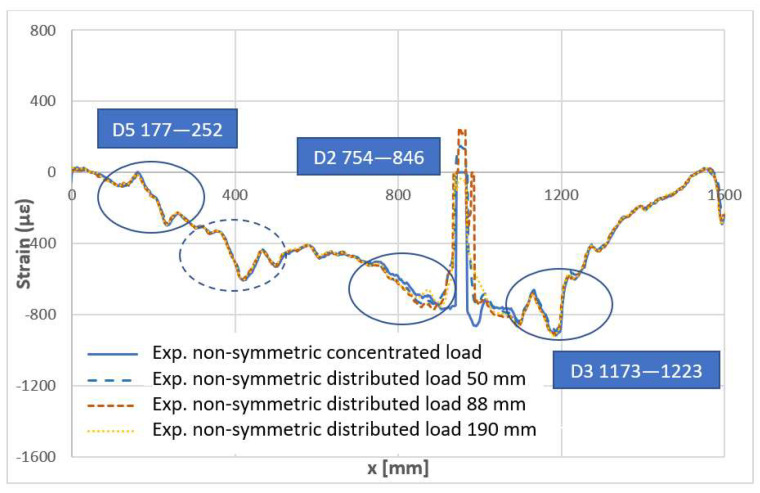
Experimental outcomes for different kinds of non-symmetric load. The measure in mm indicates the spanwise extension of the load, constant in width and covering the entire beam. Load result is always fixed at the same value.

Finally, in Figure 39, a comparison of the experimental response of the structure after the incidence of loads of the same magnitude, but distributed over different regions, is reported. It should be remarked that different load distributions should nominally give rise to different structural responses. However, unless this region length is smaller than the correspondent beam size, it could be assumed that such a dissimilarity is contained within a small range. This easy consideration is confirmed by the curves reported in the picture. A significant variation does appear only in proximity of the load itself, which maintains its characteristic in causing a singularity in the fiber optics, but becoming smoother and smoother as it is more and more distributed. The criticality is evident: as the load is smoother, its region of influence amplifies, and it overlaps the area of the defect, making it less visible. In this latest graph, the positions of the flaws are evidenced with a continuous ellipse. A shape in the left side of the signal can be observed, very similar to the one on the right, corresponding to the D3 flaw. This irregularity, evidenced with a segmented ellipse in Figure 39, may be an index of a further damaged region.

Further considerations may be made concerning an apparent paradox that emerged from the analysis. On one hand, the damage effect, spanwise, seems to be larger than its size, with factors that approximate a factor ranging from 2 to 3 for characteristic values (tens of cm). On the other hand, the damage effect seems to vanish rapidly enough cap-wise, as confirmed from both the results on the simple coupon, and the results on the composite beam. This contrasting outcome may find an explanation on the analysis of two factors, very different in the investigated cases: the size of the flaw vs. the characteristic size of the structure in the homogeneous direction; and the main excitation path vs. the amount of decay along different directions. Both these aspects deserve dedicated studies.

## 11. SHM Logic

The aforementioned studies highlighted the possibility of detecting bonding failures by investigating the differences between strains in damaged and undamaged conditions or individuating the potential points of faults start as the ones related to large variations. An SHM algorithm (L.H.E.O., local high-edge observer), formerly developed, is herein tested. Such a tool aims to detect flaw size and location without the need of reference loads and strain signatures. In other words, its architecture is based on the large strain signal scattering generated by a bonding irregularity. Scattering is processed through an edge detection technique, similar to the ones typically used in image processing, which refer to first-order derivatives [43]. It is assumed that such a dispersion can be observed both in time and space; in other words, by referring to the evolution of the signal in time, and the difference of signals among proxy sensors. This approach is specifically devoted to the analysis of experimental signals but can be suitably adapted to manage numerical information. To limit the effects of background random noise that may hide small deviations, generating a sort of threshold of readability, cross-correlation functions are implemented on the signals, improving the signal quality [44]. Since strain deviations may be small, it is necessary to have a targeted processing tool. Deviation index features (DIF) is a methodology based on deviation features of data extracted from a strain sensor array. Four characteristic normalized parameters, or features, were considered in this study:

1. F1: temporal strain variation;
2. F2: space strain variation;
3. F3: temporal strain energy variation;
4. F4: space strain energy variation.

## 12. SHM Application

**Figure 40:**
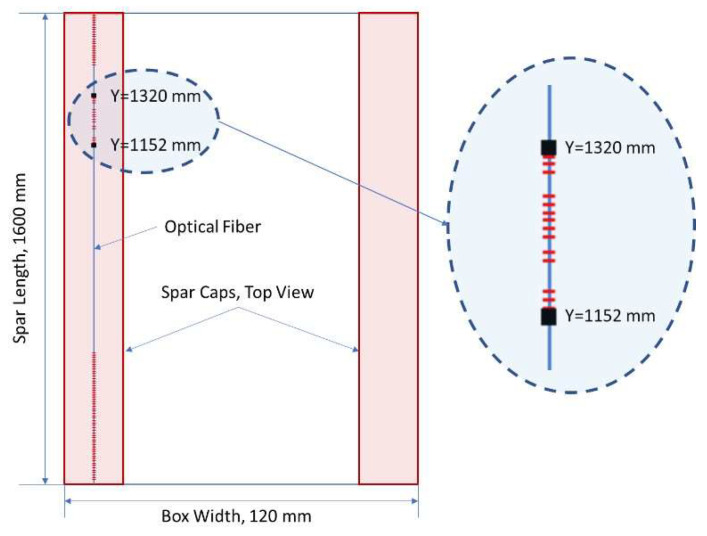
Graphical representation of test case results referred to the quadruple of loads type applied (**left**), and a magnification of the area of interest (**right**). The figure is not to scale.

**Figure 41:**
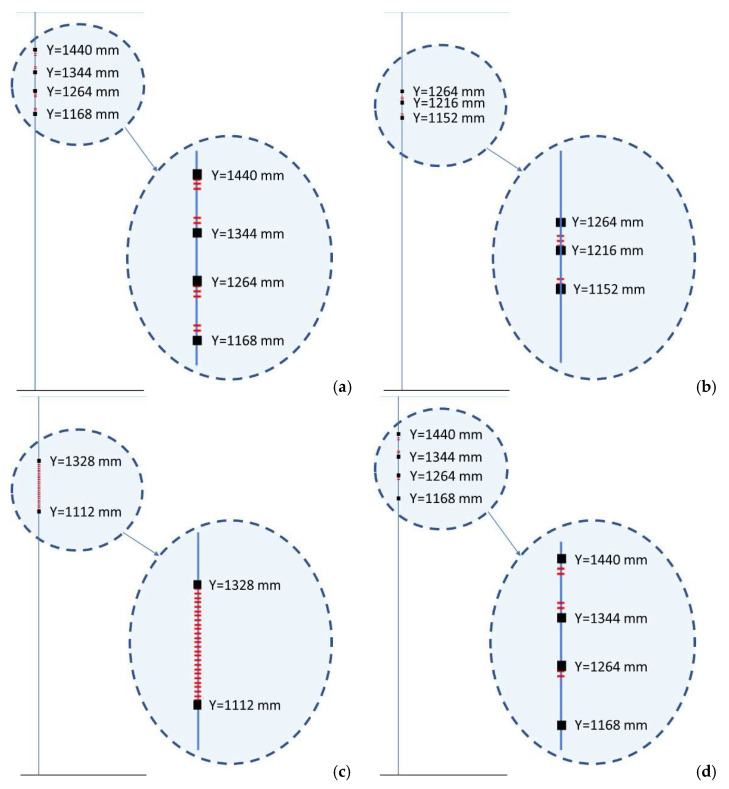
Results by referring separately to time (**a**,**c**) and space (**b**,**d**) characteristic parameters for bending 2 (**a**,**c**), and non-symmetrical excitations 4 (**b**,**d**); magnifications on the right of each picture.

Algorithm application referred to the reference structure, damaged as reported in Figure 20, under four different conditions; in fact, two more kinds of load are introduced to enlarge the available dataset (distributed load, and a combination of normal and flexural load). In total, 16 strain maps are available: four load types by two sensor arrays, deployed on the C-spar top cap within and outside the adhesive layer, by two states of the reference beam (damaged and undamaged). Since it is hard to implement time variation in numerical analysis, it was chosen to introduce the concept of variations by shifting from one set of data to another. Space variation is instead classically evaluated by considering adjacent grids. In these preliminary trials, no added disturbance is considered. The implementation of the algorithm provides in output the fault’s location and extension, as reported in Figure 40. Adhesive disconnections are merged, and the overall damage extension evaluation slightly overestimates the flaw’s length. If single load types are considered, it is confirmed that all of them provide equivalent results. In addition, the space and time features were separately considered. A graphical representation of these new outcomes, referred to as bending excitation, is reported in Figure 41. Generally, time domain features seem to perform better than space domain features. In the reported pictures, the composite beam is schematically represented as a rectangle viewed from the top. Vertical edges are then the C-spar schematized as a single line. On these lines, detected damage is reported as a series of red marks (detail is magnified on the right of each picture). Finally, in Table 7 and Table 8, a systematic comparison of the actual and predicted damage length, as well as the beginning and the end of the damage zones, is reported. 

The tables show a certain shift of the prediction with respect to the actual position of the damage, perhaps a consequence of the fact that the flaw produces an effect on a wider area than its actual dimension (Figure 16 and Figure 17). Conversely, the absolute length estimations appear to be caught, at least in the most cases, confirming the goodness of the selected approach.

## 13. Conclusions

Based on numerical computations, this paper presents a first characterization of the effects of adhesive imperfections, addressing distributed strain sensing technology. The illustrated concept is in line with current aeronautical standards and regulations and can be seen as a first step towards a wider application of bonded parts on load-critical aircraft elements. Specifically, the work reports a detailed analysis of bonding layer interruptions on a typical composite wing beam, made of several elements glued together. Symmetrical and non-symmetrical load types are investigated. Both embedded fibers, i.e., deployed within the adhesive, and external fibers, i.e., deployed on the external surface of the component spars, are considered.

In both numerical simulations and experimental tests, strain is acquired along a high-density sensor line. FE analyses permitted verification as adhesive discontinuities cause evident deviations in the deformation function with respect to the undamaged layout. The main characteristic of such changes is the appearance of point spikes, distributed over a length slightly larger than the dimension of the imposed damage. However, while for the first case, such a double-spiked shape is distributed along the whole segment affected by the fault in the guise of a rough 1-period sinusoid, in the second case, it appears as a couple of isolated peaks equally directed at the extremities. In both cases, the function between the peaks is almost linear. The strain outline is practically coincident in the undamaged and damaged configurations but at the unconformity regions. In other words, the effect of the flaw appears to vanish outside the damage location. That behavior is confirmed for both the load configurations that are herein considered.

Data are elaborated by a proprietary SHM algorithm, which takes advantage of such variability and uses finite differences, cross-correlation functions, and a suitable selection of signal characteristics to individuate critical regions by strain measurements, since irregularities could also be intrinsic in the analyzed structure (for instance, thickness variations). Of course, as far as such information is known, it may be filtered a priori. As it is, it could also represent an index of what kind of effect should be expected by architectural irregularity of certain characteristic lengths. This study also shows that sensor arrays embedded within the bonding layer are more effective in filtering structural irregularities than the ones deployed on the exterior of the beam. The authors prefer, then, to rely on embedded rather than external sensors, as they seem to filter disturbances somehow.

The experiments carried out by the authors in this and other studies show that strain evidence is barely influenced by the load position and distribution, even though it should be underlined that more complex loads give rise to more complex structural responses (i.e., strain functions), which, in turn, may lead to a less evident effect of the flaw. Such an aspect deserves more attention and further investigations. Because of that, for instance, it could be convenient to acquire a certain knowledge of the objective structure and the operation conditions in order to properly extract the flaw location and size. In that sense, a data-driven algorithm, relying on a certain knowledge of the structural response, acquired along its operation, may be convenient for setting certain discriminator values. 

Of course, these statements cannot be considered to have a general value, but are limited to the load types and structural components (full-size and full-dimension) that have been met during the cited studies. A devoted theoretical model could improve this understanding and represents one of the next steps of research. It is believed that the numerical analyses carried out up to now can somehow facilitate an appropriate formulation.

Concerning the selection of the parameters of interest, implemented in the reported SHM algorithm, strain energy and its derivative along space and time have been considered for the data elaboration and achieve a prediction of the damage presence and extension. The rationale behind that choice is that, as the numerical analyses confirmed, and as may be intuitive, flaws produce a strong strain variation, which has, however, limited and local effects. A strong strain variation is then expected to occur in proximity of the flaw’s location. So, strain and its quadratic function (a measure of the strain energy) have been considered characteristic features of the implemented SHM algorithm. It may be necessary to state that, currently, no evidence can be claimed about the fact that those parameters are the best possible ones, or the only ones that cab be considered in an SHM algorithm.

In the opinion of the authors, and according to the presented results, multi-damage detection may be possible because of the local characteristic of the damage effect. In other words, flaws create a very limited perturbation around their location, so that there is almost no interaction among different flaws in a same structural component. If and when the effects of two flaws match, they can be said to be so close to each other that the damage entities are finally merged into a single one.

Ongoing research is focused on identifying preliminary relations among fault descriptors and their real sizes. A devoted study is currently being performed to identify the characteristics of the extinction area and its functional dependences. Additional efforts are being spent to verify the algorithm capability in further, more complex case studies. All those steps are fundamental for assessing a design tool for SHM systems. The following actions are planned in the next phases of the research:

1. To use analytical modeling and experimental outcomes to draft a preliminary hypothesis of categorization and classification of different damage types;
2. To investigate different ways to model bonding between structural elements for catching their influence on SHM systems capability to identify related faults;
3. To extend this basic knowledge to further types of damage, so as to project the use of SHM systems as preliminary and fast NDI tools;
4. The design of a disbonding detection process to augment SHM systems with a methodical approach.

## Figures and Tables

**Table 1:** Comparison of distributed fiber optic technologies.

Characteristic/Type	Rayleigh	Brillouin	Raman
**Domain**	OFDR	BOTDR; BOTDA	OTDR
**Measuring Parameters**	Strain; Temperature	Strain; Temperature	Strain; Temperature
**Maximum Fiber Length**	100 m	10 km	20 km
**Spatial Resolution**	2.5–5 mm	10 cm	1–2 m
**Nominal Strain Accuracy**	1 με	25 με	1 με

**Table 2:** Composite mechanical properties (single lamina).

Property	Value
E_11_ [MPa]	134,300
E_22_ [MPa]	9700
G_12_ [MPa]	6050
G_13_ [MPa]	6050
G_23_ [MPa]	3850
υ_12_	0.34

**Table 3:** Adhesive mechanical properties.

Property	Value
E [MPa]	3272
G [MPa]	1230
Υ	0.33

**Table 4:** Characteristic parameters vs. debonding length.

Debonding Length (DL, mm)	Strain Band (SB, mm)	Delta Strain (DS = MS − mS; με)
8	32	854
16	48	1607
24	64	2466
32	80	3769
48	104	5620
64	120	7020
80	136	7673
96	152	8528
112	168	9307

**Table 5:** Characteristic parameters vs. debonding length.

Debonding Length (DL, mm)	Normalized Band (NB, -)	Normalized Delta Strain (NS; με/mm)
8	4.0	26.7
16	3.0	33.5
24	2.7	38.5
32	2.5	47.1
48	2.2	54.0
64	1.9	58.5
80	1.7	56.4
96	1.6	56.1
112	1.5	55.4

**Table 6:** Damage characteristics.

Flaw ID	Width (Cap-Wise, mm)	Length (Spanwise, mm)	Extension (Spanwise, mm)
**D2**	10	92	754–846
**D3**	20	50	1173–1223
**D5**	20	75	177–252

**Table 7:** Systematic comparison of the reported predictions (Figure 40 and Figure 41): damage length. TL = top left; TR = top right; BL = bottom left; BR = bottom right.

Reference Figure	Reference Length (mm)	Estimated Length (mm)	Absolute Difference (mm)
Figure 40	96	168	72
Figure 40	96	NA	NA
Figure 41, TL	96	96	0
Figure 41, TL	96	96	0
Figure 41, TR	96	112	16
Figure 41, TR	96	NA	NA
Figure 41, BL	96	216	120
Figure 41, BL	96	NA	NA
Figure 41, BR	96	96	0
Figure 41, BR	96	96	0

**Table 8:** Systematic comparison of the reported predictions (Figure 40 and Figure 41): damage start and finish. TL = top left; TR = top right; BL = bottom left; BR = bottom right.

Reference Figure	Damage Start and End Position (mm)	Estimated Damage Start and End Position (mm)	Absolute Difference (mm)
Figure 40	1152/1248	1152/1320 (*)	0/72 (*)
Figure 40	968/1064	NA/NA	NA/NA
Figure 41, TL	1152/1248	1344/1440	192/192
Figure 41, TL	968/1064	1168/1264	200/200
Figure 41, TR	1152/1248	1152/1264 (*)	0/16 (*)
Figure 41, TR	968/1064	NA/NA	NA/NA
Figure 41, BL	1152/1248	1112/1328 (**)	−40/80 (**)
Figure 41, BL	968/1064	NA/NA	NA/NA
Figure 41, BR	1152/1248	1344/1440	192/192
Figure 41, BR	968/1064	1168/1264	200/200

(*) In coherence with the other data, it would be possible in principle that the estimates refer to the bottom line (i.e., an upper shift is applicable to all the forecast). (**) It is possible the faults are merged in the prediction (significant excess of length estimation).

## Data Availability

Data are not available publicly for confidentiality reasons.
